# Primary Motives and Barriers to Physical Activity Participation Among Students Registered at a Semi-Rural University: A Mixed-Methods Study

**DOI:** 10.3390/ijerph22030344

**Published:** 2025-02-26

**Authors:** Silindokuhle Sanele Radebe, Gerrit Jan Breukelman, Anné S. Joubert, Lourens Millard

**Affiliations:** 1 Department of Human Movement Science, Faculty of Science, Agriculture and Engineering, University of Zululand, KwaDlangezwa 3886, South Africa; breukelmang@unizulu.ac.za (G.J.B.); millardl@unizulu.ac.za (L.M.); 2 Department of Nursing Science, Faculty of Science, Agriculture and Engineering, University of Zululand, KwaDlangezwa 3886, South Africa; jouberta@unizulu.ac.za

**Keywords:** physical activity, university students, semi-rural settings, motivations, barriers, mixed methods

## Abstract

Physical inactivity among university students remains a global health concern, especially in semi-rural settings where unique barriers such as limited facilities, cultural norms, and inadequate transportation persist. This study aimed to identify the primary motivations and barriers to physical activity participation among students at a semi-rural university, providing insights for tailored interventions. A mixed-methods explanatory sequential design was used. In the quantitative phase, 414 questionnaires were distributed across four faculties, with 328 completed responses analysed using *t*-tests and factor analysis. The qualitative phase involved semi-structured interviews with 23 students, analysed through conventional content analysis. The quantitative findings revealed key motivators for physical activity, which included enjoyment (mean = 4.51, *p* < 0.001), social interaction (mean = 3.99, *p* < 0.001), and health benefits (mean = 3.27, *p* = 0.001). However, for barriers, heavy academic workload reached statistical significance (mean = 3.88, *p* < 0.001). In contrast, qualitative data uncovered additional barriers such as insufficient facilities and poor communication, which were not captured in the quantitative phase. Factor analysis identified four constructs for motivations (e.g., coaching, social, health, enjoyment) and barriers (e.g., club processes, excuses, external factors, lack of interest). Qualitative data corroborated these findings, highlighting issues such as insufficient visibility of physical activity programs, time barriers, and inconsistent coaching quality. This study underscores the importance of addressing structural barriers, such as upgrading facilities, improving communication strategies, and enhancing coaching quality, to increase physical activity participation. By implementing strategic interventions, universities can foster inclusive environments that better support students’ well-being and engagement, ultimately promoting healthier lifestyles among university populations.

## 1. Introduction

According to the World Health Organization [1], approximately 80% of young individuals fail to achieve the minimum recommended level of physical activity, which is to accumulate at least 150 min of moderate-intensity or 75 min of vigorous-intensity physical activity per week. Physical activity is characterized as any physiological movement resulting from skeletal muscle contractions that substantially elevate energy expenditure [1]. Cardiovascular disease, hypertension, type II diabetes, osteoporosis, breast cancer, and colon cancer are all increased by physical inactivity [2]. Physical inactivity is a primary risk factor for death, contributing to approximately 5.3 million deaths annually on a global scale [3]. Conversely, there is substantial empirical evidence that indicates numerous advantages to engaging in physical activity or sports on a consistent basis, including aesthetic, psychological, and social advantages.

Physical activity offers numerous advantages for the health and well-being of an individual, such as enhanced cognitive function, sleep, and mental health, as well as a decreased risk of noncommunicable diseases [4]. In the context of academic pressures, physical activity is an essential instrument for students to maintain mental well-being and alleviate stress [5]. Furthermore, it enhances energy levels, temperament, cognitive function, stress management, learning and memory, self-image, and overall social and psychological quality of life [1,5]. In spite of these benefits, it has been reported that university populations in rural and semi-rural areas are less inclined to engage in physical activity than those in urban areas [6].

In South Africa, particularly in rural and semi-rural areas, students encounter numerous obstacles that contribute to decreased levels of physical activity. These include cultural norms that may discourage physical activity, inadequate public transportation, and limited access to facilities such as gyms, sports fields, and parks [2,6]. Additionally, recent studies highlight a concerning trend of physical inactivity among South African university students. For instance, Johannes et al. [7] reported that 29% of university students are physically inactive. These students may face increased risks of noncommunicable diseases, mental health challenges, and diminished self-esteem due to physical inactivity. These risks underscore the importance of understanding their barriers and motivations for participation in physical activity [4].

Numerous research studies have been initiated to investigate the motivations and barriers behind students’ participation in physical activity as a result of the growing awareness of the advantages of physical activity and the escalating rate of physical inactivity [5,7]. Although this subject has been extensively researched, there is a dearth of studies conducted in developing countries such as South Africa, specifically within semi-rural university settings. Existing research has predominantly focused on urban students, with limited attention given to the unique challenges faced by semi-rural students. By addressing this gap, this study aims to explore the primary motivations and barriers to physical activity participation among semi-rural university students in South Africa, a context that has been under-represented in the existing literature

## 2. Materials and Methods

This study implemented an explanatory sequential mixed-methods design, which integrates quantitative and qualitative methodologies to offer a thorough comprehension of the primary motivations and barriers to physical activity participation among university students in semi-rural areas. Table 1 presents an overview of the research design.

### 2.1. Participants

RoaSoft.com’s [9] sample size calculator http://www.raosoft.com/samplesize.html (accessed on 10 April 2024) was used to determine an initial target of 318 participants. To address potential non-response, an additional 96 participants were recruited, resulting in a total sample of 414. This calculation was based on a 95% confidence level with a margin of error of 5%, expecting 70% response distribution. Due to limited access to complete enrolment data from university administration, stratification was estimated based on gender, study level, and faculty, utilising publicly available enrolment data from the university website. The target sample sizes for each faculty were as follows: Faculty of Humanities and Social Sciences with 112 participants, Faculty of Science and Agriculture with 104 participants, Faculty of Education with 102 participants, and Faculty of Commerce, Administration, and Law with 96 participants. The sample consisted of 178 female participants, 149 male participants, and 1 participant who identified as other, with ages ranging from 18 to 35, enrolled in full-time courses at the University of Zululand (Kwadlangezwa campus). To guarantee that each faculty was represented, participants were chosen through stratified random sampling [10]. This approach allows for findings that are more reflective of the population. In an effort to gain deeper insights into the quantitative results, 23 open-ended semi-structured interviews were conducted with purposively selected students during the qualitative phase. Participants in this phase were chosen based on their prior completion of the initial questionnaire and their active participation in physical activity programs offered by the university through the Sport and Recreation Department, i.e., engaging in at least 150 min of moderate-intensity or 75 min of vigorous-intensity physical activity per week. This selection process ensured that participants provided valuable insights that were aligned with the research objectives.

#### Instruments

A self-administered questionnaire was adopted and adapted in accordance with the existing literature [11] on the participation of university students in physical activity. There were three sections in the questionnaire: Information on demographics, including age, gender, year of study, faculty, and residential status, followed by motivations for physical activity participation, was evaluated using a 5-point Likert scale to determine the extent of agreement or disagreement with the various motivations for physical activity participation. Similarly, a five-point Likert scale was implemented to assess agreement or disagreement with potential barriers to participation. The questionnaire was adapted to reflect the semi-rural university context, ensuring relevance to the South African higher education environment. Additional items on facility accessibility, coaching communication, and cultural influences were included to capture unique barriers identified in this environment. Items were selected and adapted from validated tools used in previous studies on student physical activity participation [11]. A pilot study (*n* = 20) was conducted to assess the questionnaire’s clarity; based on pilot feedback, minor wording adjustments were made for clarity and relevance. Cronbach’s alpha coefficients confirmed internal consistency (motivations scale: α = 0.81 and barriers scale: α = 0.76). Face validity was confirmed by experts in physical activity research. To further explore barriers and motivations, 23 semi-structured interviews were conducted following quantitative data analysis. Interview questions were informed by the results from the quantitative phase, with predefined themes including health benefits and social interaction. However, an inductive approach was also used, allowing new themes to emerge from participant responses. The interviews were conducted by the lead researcher privately, in person, and were audio-recorded.

### 2.2. Data Collection Process

Data collection for the study began after receiving ethical approval (UZ-REC 0691-008). The recruitment process involved collaboration with lecturers, tutors, mentors, fitness instructors, and sports coaches to reach a broad student audience, as obtaining a full list of enrolled students was difficult. Invitations to participate were shared through emails, tutorials, and lectures, with an information page detailing the study’s purpose, requirements, data collection methods, and ethical considerations. Participants were asked to confirm their involvement by a specific deadline, with attention paid to maintaining proportional representation across faculties, study levels, and gender. Efforts were made to engage under-represented groups throughout the data collection period. Participants were given the study information sheet, informed consent form, and questionnaire in either English or IsiZulu. Participants signed the consent form, completed the questionnaire, and returned it to the researcher. A unique code was assigned to each response for tracking. The researcher distributed the questionnaires in lecture halls, tutorials, and training sessions. The questionnaires took approximately 10–15 min to complete, with some being returned the following day. A purposive subset of participants was selected for follow-up interviews after the quantitative data analysis, with participants’ written consent interviews conducted in English and each interview lasting between 15 and 35 min.

### 2.3. Data Analysis

Out of 414 questionnaires distributed, 328 complete questionnaires were received and analysed. The quantitative data were analysed using the Statistical Package for Social Sciences (SPSS) version 25 [12]. Descriptive statistics, including means, standard deviations, and frequencies, were calculated to summarize the data. A one-sample *t*-test [12,13] was implemented to evaluate the degree of agreement or disagreement with the enumerated barriers and motivations with respect to a neutral score of 3 on the 5-point Likert scale. To identify the fundamental constructs, the barriers and motivations scales were subjected to factor analysis with promax rotation [14]. Items with factor loadings below 0.4 were excluded. Four critical factors were identified in the factor analysis for both barriers and motivations, which accounted for a significant portion of the variance in the data. In order to confirm that the data were suitable for factor analysis, Bartlett’s test of sphericity was implemented, and the Kaiser–Meyer–Olkin (KMO) test was administered to ensure that the sampling was adequate [14]. Furthermore, independent samples *t*-tests [15] were implemented to compare the mean levels of physical activity participation, perceived barriers, and motivations between male and female students, allowing us to identify gender-based differences in these variables.

Analysis of the qualitative data was conducted through conventional content analysis [8,16]. While initial themes were informed by the quantitative findings, an inductive approach was also employed, allowing additional themes to emerge from the data [16]. Audio recordings from interviews were transcribed verbatim. The researcher was not familiar with content analysis and any qualitative software, which inhibited their ability to determine where to begin. The researcher reviewed the article “A Hands-on Guide to Content Analysis” by Erlingsson and Brysiewicz [16,17]. The article was employed as a guide, and the researcher adhered to the procedure as outlined in the article, initially manually coding and subsequently coding with Nvivo 14 [8]. Motivations (e.g., health, enjoyment, social interaction) and barriers (e.g., lack of facilities, poor communication) were the basis for the development of codes, inspired by the main themes identified in the quantitative results. Additionally, emergent themes in the qualitative data were also used to generate new codes. Content analysis enabled the identification of patterns and provided a more profound understanding of the factors that influence physical activity participation. The quantitative phase identified primary themes in barriers and motivators, which then informed specific questions in the qualitative interviews. This sequential design enabled deeper exploration into initial questionnaire findings and added context for understanding student feedback.

## 3. Results

### 3.1. Quantitative Findings

The majority of respondents (53%) who were within the age category of eighteen to thirty-five were female. The racial representation of the sample was Black and Indian participants, with no White students present in the sample. The study sample consisted of South African (99%) and international students (1%) who were enrolled in the University of Zululand’s Faculty of Sciences, Agriculture, Engineering (27.5%), Faculty of Education (28.1%), Faculty of Commerce, Administration and Law (23.1%), and Faculty of Humanities and Social Sciences (21.3%). More than forty percent (42.2%) of the sample identified themselves as non-participants, while the majority (57.8%) were active participants in physical activities.

#### 3.1.1. Barriers to Physical Activity Participation

A one-sample *t*-test was conducted to assess the level of agreement or disagreement with the various barriers to participation in physical activity among students. The average agreement score for each barrier was tested against a central score of 3. A score significantly higher than 3 (*p* < 0.05) indicates significant agreement with the barrier, whereas a score significantly lower than 3 indicates significant disagreement. If the result is not significant (*p* ≥ 0.05), this indicates that there is neither significant agreement nor disagreement with the barrier. The results are presented in Table 2 below

The results indicate significant agreement among students that excessive academic workload contributes to their lack of physical activity (mean = 3.88, *p* < 0.001). Conversely, there is significant disagreement regarding several other reasons for their non-participation in physical activities. These reasons include taking medication, the exacerbation of allergies, disabilities that render facilities inaccessible, evening work commitments to support their studies, personal beliefs that prevent participation, challenges in joining teams (especially for non-South African students), concerns about non-UNIZULU students being prioritized for team selection, and difficulties in communication due to language barriers with coaches. Additionally, there is no significant agreement or disagreement concerning reasons such as lacking athletic ability, fear of participation, not having a partner to engage with, unawareness of available campus activities, and the high costs of gym memberships being a challenge.

To understand the underlying structure of these barrier perceptions, an exploratory factor analysis with promax rotation was conducted, and the analysis yielded four primary constructs that together accounted for 40.72% of the variance. The constructs included Club Processes (19.9% variance, Cronbach’s alpha = 0.817), which encompassed issues such as disorganization, late coaches, unfair team selection, and a lack of qualified coaches, demonstrating high internal consistency; Lack of Interest (9.6% variance, Cronbach’s alpha = 0.721), which was associated with a general disinterest in physical activities, such as a lack of enjoyment or not considering oneself a “sports person”; Excuses (6.3% variance, Cronbach’s alpha = 0.623), which encompassed personal justifications such as a lack of awareness of activities or a lack of a training partner; and External Reasons (5.0% variance, Cronbach’s alpha = 0.549), which included obstacles such as health conditions, work commitments, or personal beliefs. Subsequent one-sample *t*-test results revealed significant disagreements with all four factors, Club Processes (M = 2.13, *p* < 0.001), Lack of Interest (M = 2.70, *p* < 0.001), Excuses (M = 2.82, *p* = 0.014), and External Reasons (M = 1.80, *p* < 0.001). These findings suggest that students do not perceive these factors as significant barriers to physical activity, with Club Processes and External Reasons showing the strongest disagreements. Complete tables including item factor loadings (Table S1), variance (Table S2), and one-sample *t*-test results for each factor (Table S3) are provided in the Supplementary Tables.

#### 3.1.2. Motivations for Physical Activity Participation

A one-sample *t*-test was performed to evaluate the level of agreement or disagreement regarding the motives for participating in physical activity among students. The average agreement score for each motive was compared against a central score of 3. A score significantly higher than 3 (*p* < 0.05) indicates strong agreement with the motive, while a score significantly lower than 3 signifies strong disagreement. In cases where the result is not significant (*p* ≥ 0.05), this suggests that there is neither significant agreement nor disagreement regarding that specific motive. This statistical approach provides insight into which factors students view as motivating influences for their engagement in physical activities. The results are presented in Table 3 below.

The analysis demonstrates that students were in strong agreement with several motivational items that influence their engagement in physical activity. These items include the health benefits associated with exercise (m = 4.52), the reduction in stress resulting from academic workloads (m = 4.51), the enjoyment of physical activities (m = 4.47), the enhancement in self-image (m = 4.57), the accessibility of facilities, opportunities for social interaction, and effective communication from coaches. Conversely, there was substantial disagreement regarding certain motivators, including the lack of alternative activities and the participation of companions. Furthermore, no significant differences were noted for items like the merit-based selection of university team players, concerns about injury, or recommendations from health professionals. Overall, the results suggest that students generally recognize the positive impact of physical activity on their health, stress levels, and overall motivation and happiness.

Factor analysis identified four primary motivational constructs driving students’ participation in physical activity, accounting for 51.30% of the variance. The constructs included Coaching and Communication (24.0% variance, Cronbach’s alpha = 0.800), emphasizing the importance of well-planned and communicative coaching; Social Interaction (12.6% variance, Cronbach’s alpha = 0.808), highlighting the value of meeting new people and learning about diverse backgrounds; Health (8.3% variance, Cronbach’s alpha = 0.704), which emphasized the health benefits of physical activity; and Enjoyment (6.4% variance, Cronbach’s alpha = 0.576), which focused on personal enjoyment and stress reduction. One sample *t*-test confirmed significant agreement with all four constructs, with *p*-values less than 0.001 for each. These results underscore the significant role of coaching quality, social interactions, health benefits, and enjoyment in motivating students to engage in physical activities, suggesting that effective programs should incorporate these elements to enhance student participation. Detailed tables presenting item factor loadings (Table S4), variance explained (Table S5), and one-sample *t*-test results (Table S6) for each motivational factor are available in the Supplementary Tables.

Given the known influence of gender on physical activity participation, we examined gender differences in motivations, barriers, and participation frequency using independent samples *t*-tests. The results reveal that males reported significantly higher agreement than females regarding motivations for physical activity. Specifically, males (mean = 4.12) were more motivated by social reasons than females (mean = 3.79), with a significant difference (t (179) = 2.374, *p* = 0.019). Similarly, for health-related motivations, males expressed greater agreement (mean = 3.40) compared to females (mean = 3.07), with t (179) = 2.110, *p* = 0.036. However, independent samples *t*-tests found no significant gender differences in perceived barriers to physical activity (*p* > 0.05), suggesting that both genders experienced similar barriers. Regarding participation frequency, a greater proportion of males (70.6%) engaged in physical activity four to five times per week compared to females (55.4%). Fisher’s Exact Test confirmed a significant relationship between gender and participation frequency (11.533, *p* = 0.007), indicating that males are generally more physically active than their female counterparts.

### 3.2. Qualitative Findings

Using semi-structured interviews, qualitative insights were obtained in the present study to facilitate a more profound comprehension of the participants’ perspectives and experiences. The objective of these interviews was to either support or refute the statistical results. The qualitative findings suggest that physical activity participation emerges as a valuable stress-relieving mechanism in the university context.

“*I joined to relieve stress during exams and to make friends*”.

(*Que322*; *Que325*)

“*Participating helps with my mental health; when I’ve had a stressful day, I go to practice, and I feel better*”.

(*Que324*; *Que328*).

The above quotes illustrate the mental health advantages that students gain from participating in physical activities. This strongly supports the quantitative finding “It reduces the stress of too much academic work” (mean = 4.51, *p* < 0.001). The social benefits of sports are evident in statements like

“*Sports unite us; I can say I have brothers for life*”.

(*Que254*; *Que264*; *Que269*).

“*It’s like a home away from home… you get to express yourself, get away from things*”.

(*Que286*; *Que328*).

Such sentiments align with the quantitative result “I get to meet people and make new friends” (mean = 3.99, *p* < 0.001), underscoring the role of sports in fostering social connections. However, these themes are often undermined by time barriers due to academic obligations, which are significant barriers to participation. Many students expressed concerns such as

“*We don’t have time to do sports because we’re so busy with our studies*”.

(*Que001*; *Que178*; *Que262*),

Which strongly supports the quantitative finding “I have an excessive amount of academic work” (mean = 3.88, *p* < 0.001). These results suggest that students face challenges in balancing academic responsibilities with extracurricular engagement. A lack of awareness regarding available sports opportunities was a recurring theme.

“*Students don’t know how to join or where to go to join certain sports codes*”.

(*Que001*; *Que269*; *Que327*; *Que328*).

These findings align with the questionnaire item “I am unaware of the activities that are available on my campus” (mean = 2.90, *p* = 0.349), which, despite a non-significant *p*-value, reflects a general lack of awareness. This lack of promotional communication particularly affects first-year students, as evidenced by the low reported participation rate of 33.7% among this group. Access to sports facilities is both a motivator and a challenge for students. While the questionnaire finding “The facilities are easily accessible” (mean = 3.81, *p* < 0.001) suggests that most students perceive facilities as accessible, qualitative data revealed logistical challenges. For example, sharing facilities with other groups creates scheduling conflicts and overcrowding.

“*The lighting is a big issue for us, especially since it gets dark earlier in the winter*”.

(*Que001*; *Que319*; *Que324*.)

Additionally, inadequate lighting in facilities was noted as a concern.

“*We share the gymnasium with dancers, aerobics and karate students, which limits practice time*”.

(*Que318*; *Que327*).

These issues underscore the need for improved resource management, better scheduling, and facility upgrades to ensure equitable and safe access for all users. The availability and quality of coaching staff revealed a complex picture. Some students appreciated their coaches, while others expressed dissatisfaction:

“*We don’t have enough qualified coaches, and often the team captain has to take over, which isn’t ideal*”.

(*Que269*; *Que322*; *Que328*).

“*The coaches that we have aren’t qualified and I heard they aren’t even paid*”

(*Que200*; *Que262*; *Que286*).

These qualitative findings directly contradict the questionnaire response “Coaches are knowledgeable and competent” (mean = 3.99, *p* < 0.001), suggesting that coaching quality varies across sports programs. This inconsistency acts as both a motivator and a barrier. In some cases, team captains reportedly took on coaching responsibilities due to the lack of qualified staff.

## 4. Discussion

The results of this study are slightly higher but still consistent with the study of Mthethwa [11] at the University of KwaZulu-Natal (UKZN) where 40% of students did not participate in any physical activity. Similar trends of low engagement are noted in other institutions. A recent study conducted by Alkhawaldeh and colleagues revealed that only 48.1% of university students reported engaging in physical activity [18]. This trend is concerning given that studies have clearly documented the benefits of regular physical activity for both mental and physical health, particularly for students navigating academic pressures. Understanding barriers such as heavy academic workloads, insufficient facilities, and poor communication is crucial for developing effective strategies to encourage greater involvement in physical activities among university students.

### 4.1. Barriers to Physical Activity Participation

The findings of this study reveal that students in this study expressed slight disagreement with the notion that their participation is impeded by constructs of a lack of interest and excuses/justifications. This suggests that personal disinterest or justifications are not the primary reasons for students to not engage in physical activity among this population. In contrast, the findings of a similar study conducted in 2021 at the University of Venda indicated that more than half of the participants (53%) reported laziness (lack of interest) as a limiting factor for physical activity participation [19]. Even though this was not a limiting factor for this population, it is important to acknowledge findings from previous studies. Research has shown that additional factors, such as self-efficacy (belief in one’s ability to successfully participate in physical activities), also influence student participation [20]. Certain students may experience feelings of inadequacy or incompetency, which can ultimately hinder their participation in structured activities. Moreover, students may be discouraged from participating in specific physical activities, particularly those that involve high levels of physical exertion, due to their apprehension regarding injury [7]. The students’ strong disagreement with the notion that factors such as religious or cultural commitments were significant barriers was evident in the analysis of external reasons. However, prior research has underscored the significance of encouragement from family, friends, and colleagues in encouraging students to engage in physical activities or sports [7]. Additionally, the results of this investigation are at odds with those of Mathebula [19], in which female students identified a scarcity of companions as the primary factor contributing to their non-participation. In the absence of robust social encouragement, students who are new to campus or lack established peer networks may experience feelings of isolation or lack of motivation to participate in activities [20,21].

The analysis identified time barriers as a substantial structural barrier, particularly in the context of heavy academic workload. This was also reflected in the qualitative section, where participants expressed that they do not have time as they are busy with their studies. Mthethwa [11] reported that a comparative investigation of structural barriers among students at the UKZN indicated that both participants and non-participants were confined by time and schedule because students were overburdened with academic work. This interpretation is also consistent with the findings of another study which highlighted barriers to participation stemming from a lack of awareness about physical activity, time barriers due to academic obligations, and financial barriers [22]. It is noteworthy to observe that, while respondents admit that they have too much academic work, most respondents still find time in their demanding academic schedules to participate in physical activities. This reinforces Mugwedi and Mulibana’s [23] suggestion that university students should be taught time management skills to overcome these institutional barriers. Furthermore, another study conducted at a university in Botswana highlighted that academic burden and deteriorated facilities were identified as significant obstacles to physical activity [24]. Although club processes were not perceived as significant barriers in this study, many students may still face challenges that were not directly explored by the questionnaire but were later captured in the interview such as a lack of awareness about opportunities to participate in physical activity, limitations in facility scheduling or access to facilities, and the availability of facilities. The qualitative findings revealed that a significant issue was the lack of awareness about available sporting opportunities, particularly among first-year students. The qualitative findings also highlighted insufficient promotional communication. This gap likely contributes to the low participation rate of 33.7% among first-year students who suggested that sports activities be introduced during orientation. Meanwhile, access to facilities were perceived as both motivations and challenges; while most students found facilities generally accessible, logistical issues such as shared usage and inadequate lighting created barriers. These findings underscore the need for enhanced communication strategies, resource management, and infrastructure upgrades to improve awareness and equitable access. All these findings are consistent with the existing literature which frequently identifies time and financial barriers as significant obstacles, as well as limited access to facilities, an insufficient number of facilities, a lack of resources, and equipment that is too old [7,11,19,21].

### 4.2. Motivations for Physical Activity Participation

The analyses identified personal health, enjoyment, and stress relief as significant motivators. The most significant factor as reported by the *t*-test results was enjoyment. The students reported that physical activity improved their overall demeanour and provided an enjoyable relief from academic stress. Similarly, health-related motivations were also observed. Students acknowledged that their participation reduced their dependence on medication and reduced the risk of non-communicable diseases. The results are consistent with the findings of Ebben and Brudzynski [25], which indicated that the most prevalent motivations for sports participation were general health, stress reduction, enjoyment, and feeling good. Johannes et al. [7] also found that students are primarily motivated to engage in physical activity for their personal health and psychological well-being. These findings support the notion that participation in physical activities can contribute to improved mental health in students [21].

Social motivations are also substantial factors in the determination of participation. It was reported by students that engaging in physical activities is driven by the desire to make new acquaintances, interact with peers from diverse backgrounds, and meet new people. This supports the notion that physical activity serves as a social platform, fostering social integration and community among students [24,25]. Students establish meaningful alliances through physical activities, which alleviate the stress associated with the challenges of university life and the heavy academic workloads. Another study found that university students are more motivated to participate in physical activity when their family and acquaintances are present during the activities [26,27]. The formation of these meaningful friendships enables a student to enjoy the experience with someone close and changes the individual situation into a learning adventure with meaning from which one could hopefully grow [26]. A similar study found that physical activity facilitators included social support from friends and family (for social interaction, providing motivation and encouragement), social media (for relatability and motivational purposes), and gaining recognition from others [7]. All these findings demonstrate that the level of physical activity exhibited by a student is significantly influenced by the encouragement of their mentors and peers. If mentors or peers encourage them to engage in regular physical activity during their tertiary years, they are anticipated to make informed decisions regarding their physical activity participation to enhance their overall well-being.

Coaching and communication constructs emerged as substantial structural motivators for student participation in physical activity. Professionalism, punctuality, and effective communication from coaches were highlighted as essential for a positive experience. Students emphasized the importance of well-organized sessions, accessible equipment, and well-maintained facilities. While some students found well-structured coaching motivating, others expressed frustration with inconsistent or absent coaches, undermining the overall positive impact.

To further investigate gender disparities in physical activity participation, barriers, and motivations, independent samples *t*-tests were conducted. The analysis confirmed significant gender differences in participation frequency, with 70.6% of males engaging in physical activity four to five times per week, compared to 55.4% of females. However, no significant gender differences were observed regarding barriers to physical activity, including club processes, a lack of interest, excuses, or external barriers (*p* > 0.05), suggesting that both males and females perceive these obstacles similarly. In contrast, when examining motivations, males reported significantly higher agreement for participating in physical activities due to social reasons (*p* = 0.019) and health-related benefits (*p* = 0.036) compared to females. These findings contradict the study by Alkhateeb et al. [28] which suggests that females are more inclined to engage in physical exercise for social reasons compared to males. However, the results of the current study align with previous findings from Mthethwa [11] which indicate that females tend to be less motivated to engage in physical activity compared to their male counterparts.

Overall, these findings emphasize the importance of tailoring interventions to enhance female students’ engagement in physical activity, particularly by addressing motivation-related factors. This study highlights the critical role of enjoyment, health benefits, and social interactions in motivating physical activity participation while identifying academic workload and structural barriers as primary barriers. To enhance student participation, universities should prioritize facility upgrades, effective communication strategies, and qualified coaching staff while integrating physical activity into academic support programs.

## 5. Study Strengths and Limitations

One of the strengths of this study is its mixed-methods approach, which combined quantitative and qualitative data to offer a comprehensive understanding of the barriers and motivations related to physical activity among semi-rural university students. The internal validity was also strengthened by a robust sample size of 328 participants, exceeding the minimum required for reliable results. This allowed for context-based insights into the unique experiences of this population, addressing a significant gap in the literature. By focusing specifically on students in a semi-rural setting, this study contributes valuable information for developing more effective, targeted strategies to promote physical activity in such contexts.

However, the study’s scope was limited by its cross-sectional design, which captured data at a single point in time and did not account for changes in motivations or barriers over time. Additionally, this study primarily focused on institutional factors, potentially overlooking external influences such as work, family obligations, or mental health, which could also significantly affect students’ participation in physical activity.

## 6. Conclusions

The aim of this study was to investigate the primary motivating influences and barriers to physical activity participation among students at a semi-rural university. The findings of this study highlight that physical activity participation among university students is significantly influenced by various motivations. Notably, enjoyment, social interaction, and personal health benefits were identified as key motivators, emphasizing the importance of making physical activity both pleasurable and beneficial for student well-being. Furthermore, the role of social connections in physical activity participation cannot be overlooked. The findings suggest that promoting group-based activities and fostering a sense of community can increase participation rates through strong interpersonal motivations. This aligns with existing research suggesting that university experiences can be enhanced, and academic stress can be alleviated by encouraging social engagement through physical activities.

The study’s findings contribute to the broader understanding of how personal and social factors influence physical activity, supporting the idea that integrating physical activity into student life can enhance overall well-being.

Future research should explore a wider array of factors influencing physical activity, including family dynamics, environmental influences, academic achievement, mental health, and eating habits. Investigating these aspects will aid in the development of targeted interventions to enhance student participation in physical activity. Longitudinal studies are also recommended to track behavioural changes over time, providing a deeper understanding of these factors.

Moreover, employing diverse data collection methods such as experimental designs, observational studies, and focus groups alongside traditional surveys and interviews will enrich the data. Expanding research to include other South African universities will improve the generalisability of the findings, offering insights into how personal characteristics influence physical activity across different contexts. By integrating a variety of research designs and mixed-methods approaches, future studies can offer more comprehensive insights and inform more targeted interventions. There is a need for specialized solutions that consider how these factors may vary across different cultural and environmental contexts.

## Figures and Tables

**Table 1:** Design for explanatory sequential study.

Phase	Description	Data-Collection Methods	Data Analysis
Phase 1: Quantitative	A questionnaire was conducted with 328 students from four faculties to assess physical activity participation, motivations, and barriers.	Likert scales; Questionnaire; Stratified random sampling	Descriptive statistics (mean, standard deviation) Inferential statistics (e.g., *t*-tests, Chi-Square tests) Factor analysis for motivations and barriers
Phase 2: Qualitative	Twenty-three semi-structured interviews were conducted to allow students to make recommendations about current sport and recreation service delivery based on their experiences.	Open-ended semi-structured interviews; Purposive sampling	Conventional qualitative content analysis technique [8] (coding, theme generation) NVivo used for coding and organizing themes
Integration Phase	Combine quantitative results with qualitative insights. To explore how students’ recommendations align with trends identified in the quantitative phase.	Cross-referencing of quantitative results and qualitative themes	Interpretation of how qualitative data explain, contradict, or support quantitative trends

**Table 2:** Quantitative findings on participation barriers.

I do not Engage in Physical Activity Because…	n	Mean	Standard Deviation	t	df	*p*-Value
I am taking medication	134	1.65	0.886	−17.639	133	<0.001
I am not a sport person	132	2.98	1.281	−0.204	131	0.839
I have allergies, and taking part will aggravate them	131	1.76	0.904	−15.758	130	<0.001
I do not know the benefits of participating to my health	134	2.30	1.090	−7.449	133	<0.001
I have an excessive amount of academic work	130	3.88 *	1.132	8.911	129	<0.001
My disability makes facilities inaccessible	129	1.50	0.849	−20.017	128	<0.001
I do not enjoy physical activities	132	2.45	1.168	−5.441	131	<0.001
I am not interested in the activities available	130	2.66	1.191	−3.239	129	0.002
I am afraid of getting hurt	133	2.97	1.381	−0.251	132	0.802
I do not have anyone to participate with	134	2.99	1.268	−0.136	133	0.892
My friends do not participate, so I do not either	133	2.71	1.335	−2.469	132	0.015
I work in the evenings to pay for my studies	133	1.98	1.138	−10.363	132	<0.001
I cannot tolerate it when others criticise me	133	2.60	1.403	−3.275	132	0.001
My beliefs do not allow me to participate	133	1.59	0.835	−19.415	132	<0.001
I do not know what activities are available on my campus	135	2.90	1.283	−0.939	134	0.349
The equipment and resources are not in a usable condition	134	2.37	1.161	−6.249	133	<0.001
Gym membership fees are too expensive	131	2.90	1.329	−0.854	130	0.394
I do not get selected into the university teams I train for	132	2.28	1.213	−6.819	131	<0.001
People who are not UNIZULU students get picked in the teams ahead of me	132	1.91	0.842	−14.883	131	<0.001
To get into the team is difficult, especially if you are not South African	132	1.95	0.894	−13.537	131	<0.001
There is a shortage of knowledgeable and qualified coaches	134	2.16	0.941	−10.376	133	<0.001
Coaches are always late at training sessions	132	2.02	0.847	−13.360	131	<0.001
Coaches speak in a language I do not understand	132	1.93	0.821	−14.941	131	<0.001
Activities are disorganised	131	2.10	0.876	−11.772	130	<0.001
Clubs and teams are poorly managed	132	2.18	1.040	−9.040	131	<0.001
Some students have been assaulted and robbed during events	133	2.76	1.262	−2.198	132	0.030

* indicate the items that were statistically significant.

**Table 3:** Motivational items for physical activity participation.

I Engage in Physical Activity Because…	n	Mean	Standard Deviation	t	df	*p*-Value
It reduces my chances of relying on medication	177	3.50	1.394	4.743	176	<0.001
I have been advised by health expert to do so	182	2.94	1.326	−0.615	181	0.539
It reduces my chances of non-communicable diseases and allergies	178	3.39	1.240	4.170	177	<0.001
I know the benefits of participating to my health	183	4.52 *	0.710	28.954	182	<0.001
It reduces the stress of too much academic work	182	4.51 *	0.719	28.259	181	<0.001
The facilities are easily accessible	181	3.81	0.955	11.361	180	<0.001
I enjoy physical activities	182	4.47 *	0.741	26.812	181	<0.001
Activities offered on my campus are interesting	181	3.85	0.833	13.738	180	<0.001
I am not afraid of getting hurt	182	2.96	1.256	−0.472	181	0.637
Because my friends also participate	181	2.27	1.154	−8.502	180	<0.001
It keeps me active and makes me feel good about myself	180	4.57 *	0.799	26.315	179	<0.001
I get to meet people and make new friends	182	3.99	1.027	13.061	181	<0.001
It teaches me a lot about the diverse backgrounds that are represented in the student population	182	3.98	0.983	13.496	181	<0.001
I am well informed about what activities are available on my campus	180	3.62	1.026	8.061	179	<0.001
The equipment and resources offered are well maintained	178	3.55	0.969	7.584	177	<0.001
Gym membership fees are affordable	178	3.56	1.052	7.053	177	<0.001
The university’s teams are made up of players picked on merit	173	2.91	1.085	−1.121	172	0.264
Coaches communicate in a language that I can understand	177	4.10	1.061	13.813	176	<0.001
Coaches are punctual and deliver well planned training sessions	178	4.01	1.028	13.053	177	<0.001
Coaches and staff are knowledgeable and competent	175	3.99	.935	13.993	174	<0.001
I feel safe engaging in campus activities during the day or at night	177	3.64	1.160	7.322	176	<0.001
I have nothing else to do	178	2.05	1.021	−12.405	177	<0.001

* indicate the items that were statistically significant.

## Data Availability

The data supporting the study findings are available from the corresponding author upon request.

## Supplementary Materials

The following supporting information can be downloaded at https://www.mdpi.com/article/10.3390/ijerph22030344/s1, Table S1: Factor Loadings of Perceived Barriers to Participating in Physical Activity; Table S2: A summarised version of perceived barriers with Variance and Reliability Statistics; Table S3: Results of the *t*-test for each barrier construct; Table S4: Factor Loadings of Motivational Items for Physical Activity Participation; Table S5: Summary of Motivational Constructs, Variance, and Reliability for Physical Activity Participation; Table S6: Results of the *t*-test for each motivational construct.
