# Supplementary material for: Primary Motives and Barriers to Physical Activity Participation Among Students Registered at a Semi-Rural University: A Mixed-Methods Study
Source: Int J Environ Res Public Health. 2025 Feb 26;22(3):344. doi: 10.3390/ijerph22030344 (PMC11942136; doi:10.3390/ijerph22030344)
Supplement: Supplementary file 1 [file ijerph-22-00344-s001.zip › ijerph-3470725-supplementary.pdf]

**Barriers**

Table S1. Factor Loadings of Perceived Barriers to Participating in Physical Activity

| I do not engage because… | Factor | | | |
|---|---|---|---|---|
| | 1 | 2 | 3 | 4 |
| There is a shortage of knowledgeable and qualified coaches | .818 | | | |
| Activities are disorganised | .757 | | | |
| Coaches are always late at training sessions | .715 | | | |
| Clubs and teams are poorly managed | .664 | | | |
| To get to the team is difficult, especially if you are not South African | .552 | | | |
| Some students have been assaulted and robbed during events | .492 | | | |
| Coaches speak in a language I do not understand | .473 | | | |
| People who are not UNIZULU students get picked in the teams ahead of me | .411 | | | |
| I do not enjoy physical activities | | .807 | | |
| I am not interested in the activities available | | .757 | | |
| I am not a sport person | | .425 | | |
| I cannot tolerate it when others criticise me | | | .661 | |
| I do not have anyone to participate with | | | .558 | |
| I do not know what activities are available on my campus | | | .529 | |
| My friends do not participate, so I do not either | | | .462 | |
| Gym membership fees are too expensive | | | .400 | |
| My beliefs do not allow me to participate | | | | .568 |
| I work in the evenings to pay for my studies | | | | .524 |
| I have allergies, and taking part will aggravate them | | | | .500 |

Table S2. A summarised version of perceived barriers with Variance and Reliability Statistics

| **Factor** | **Construct** | **Items included** | **Variance extracted** | **Cronbach's alpha** |
|---|---|---|---|---|
| 1 | Club processes (CLUB) | • There is a shortage of knowledgeable and qualified coaches.<br>• Activities are disorganised. | 19.9 | .817 |

| | | | | |
|---|---|---|---|---|
| | | • Coaches are always late at training sessions.<br>• Clubs and teams are poorly managed.<br>• To get to the team is difficult, especially if you are not South African.<br>• Some students have been assaulted and robbed during events.<br>• Coaches speak in a language I do not understand.<br>• People who are not UNIZULU students get picked in the team ahead of me. | | |
| 2 | Lack of interest (INT) | • I do not enjoy physical activities.<br>• I am not interested in the activities available.<br>• I am not a sport person. | 9.6 | .721 |
| 3 | Excuses (EXC) | • I cannot tolerate it when others criticise me.<br>• I do not have anyone to participate with.<br>• I do not know what activities are available on my campus.<br>• My friends do not participate, so I do not either.<br>• Gym membership fees are too expensive. | 6.3 | .623 |
| 4 | External reasons (EXT) | • My beliefs do not allow me to participate.<br>• I work in the evenings to pay for my studies.<br>• I have allergies, and taking part will aggravate them. | 5.0 | .549 |

Table S3. Results of the t-test for each construct

| Barriers | n | Mean | Standard deviation | t | df | p-value |
|---|---|---|---|---|---|---|
| Club processes (CLUB) | 134 | 2.1260 | .63579 | -15.913 | 133 | <.001 |
| Lack of interest (INT) | 134 | 2.6990 | .97343 | -3.579 | 133 | <.001 |
| Excuses (EXC) | 135 | 2.8226 | .82778 | -2.490 | 134 | .014 |
| External reasons (EXT) | 135 | 1.7963 | .74693 | -18.724 | 134 | <.001 |

Table S4. Factor Loadings of Motivational Items for Physical Activity Participation

| Motives | Factor | | | |
|---|---|---|---|---|
| | 1 | 2 | 3 | 4 |
| Coaches are punctual and deliver well planned training sessions | .975 | | | |
| Coaches and staff are knowledgeable and competent | .798 | | | |
| Coaches communicate in a language that I can understand | .668 | | | |
| I am well informed about what activities are available on my campus | .387 | | | |
| It teaches me a lot about the diverse backgrounds that are represented in the student population | | .829 | | |
| I get to meet people and make new friends | | .823 | | |
| It reduces my chances of a non-communicable diseases and allergies | | | .787 | |
| It reduces my chances of relying on medication | | | .657 | |
| I have been advised by health expert to do so | | | .523 | |
| It keeps me active and makes me feel good about myself | | | | .659 |
| I enjoy physical activities | | | | .588 |
| It reduces the stress of too much academic work | | | | .406 |

Table S5. Summary of Motivational Constructs, Variance, and Reliability for Physical Activity Participation

| **Factor** | **Construct** | **Items included** | **Variance extracted** | **Cronbach's alpha** |
|---|---|---|---|---|
| 1 | Coaching and communication (MOT_CC) | • Coaches are punctual and deliver well planned training sessions.<br>• Coaches and staff are knowledgeable and competent.<br>• Coaches communicate in a language that I can understand.<br>• I am well informed about what activities are available on my campus. | 24.0 | .800 |

| | | | | |
|---|---|---|---|---|
| 2 | Social (MOT_SOC) | • It teaches me a lot about the diverse backgrounds that are represented in the student population.<br>• I get to meet people and make new friends. | 12.6 | .808 |
| 3 | Health (MOT_HEALTH) | • It reduces my chances of a non-communicable diseases and allergies.<br>• It reduces my chances of relying on medication.<br>• I have been advised by health expert to do so. | 8.3 | .704 |
| 4 | Enjoyment (MOT_ENJ) | • It keeps me active and makes me feel good about myself.<br>• I enjoy physical activities.<br>• It reduces the stress of too much academic work. | 6.4 | .576 |

Table S6. Results of the t-test for each motivational construct

| Constructs | n | Mean | Standard deviation | t | df | p-value |
|---|---|---|---|---|---|---|
| Coaching and communication (MOT_CC) | 180 | 3.9218 | .80304 | 15.400 | 179 | <.001 |
| Social (MOT_SOC) | 182 | 3.9890 | .92084 | 14.489 | 181 | <.001 |
| Health (MOT_HEALTH) | 182 | 3.2665 | 1.06494 | 3.376 | 181 | <.001 |
| Enjoyment (MOT_ENJ) | 184 | 4.5109 | .56743 | 36.118 | 183 | <.001 |
